# Coherence and measurement in quantum thermodynamics

**DOI:** 10.1038/srep22174

**Published:** 2016-02-26

**Authors:** P. Kammerlander, J. Anders

**Affiliations:** 1 Institute for Theoretical Physics, ETH Zurich, 8093 Zurich, Switzerland; 2 Physics & Astronomy, University of Exeter, Exeter EX4 4QL, UK

## Abstract

Thermodynamics is a highly successful macroscopic theory widely used across the natural sciences and for the construction of everyday devices, from car engines to solar cells. With thermodynamics predating quantum theory, research now aims to uncover the thermodynamic laws that govern finite size systems which may in addition host quantum effects. Recent theoretical breakthroughs include the characterisation of the efficiency of quantum thermal engines, the extension of classical non-equilibrium fluctuation theorems to the quantum regime and a new thermodynamic resource theory has led to the discovery of a set of second laws for finite size systems. These results have substantially advanced our understanding of nanoscale thermodynamics, however putting a finger on what is genuinely *quantum* in quantum thermodynamics has remained a challenge. Here we identify information processing tasks, the so-called *projections*, that can only be formulated within the framework of quantum mechanics. We show that the physical realisation of such projections can come with a non-trivial thermodynamic work only for quantum states with coherences. This contrasts with information erasure, first investigated by Landauer, for which a thermodynamic work cost applies for classical and quantum erasure alike. Repercussions on quantum work fluctuation relations and thermodynamic single-shot approaches are also discussed.

When Landauer argued in 1961 that any physical realisation of erasure of information has a fundamental thermodynamic work cost he irrevocably linked thermodynamics and information theory^1^,^2^,^3^,^4^,^5^,^6^,^7^,^8^,^9^. A practical consequence of this insight is that all computers must dissipate a minimal amount of heat in each irreversible computing step, a threshold that is becoming a concern with future computer chips entering atomic scales. The treatment of general *quantum* information processing tasks within the wider framework of quantum thermodynamics has only recently begun^13^. Quantum mechanics differs from classical mechanics in at least three central aspects: the special nature of measurement, the possibility of a quantum system to be in a superposition and the existence of quantum correlations. The thermodynamic energy needed to perform a (selective) measurement has been investigated^10^ and the total work for a closed thermodynamic measurement cycle explored^11^. The catalytic role of quantum superposition states when used in thermal operations has been uncovered^12^ and it has been shown that work can be drawn from quantum correlations^13^,^14^ in a thermodynamic setting, see Fig. 1. In particular, del Rio *et al.*^14^ showed that contrary to Landauer’s principle, it is possible to *extract* work while performing erasure of a system’s state when the system is correlated to a memory. This can occur if and only if the initial correlations imply a negative conditional entropy, a uniquely quantum feature. The thermodynamic process does however now require operation on degrees of freedom external to the system, i.e. the memory’s.

## Results

### Projections and the optimal work value of removing coherences

Our motivation is here to shed light on the implications of performing a measurement on a quantum state that has coherences. We will consider this task in the thermodynamic setting of Landauer’s erasure, involving a heat bath at fixed temperature *T* and operation on *N* → ∞ uncorrelated and identically prepared copies of the system (i.i.d. limit). This is of interest in the context of the quantum Jarzynski equality, for example, and will also be central for experiments testing quantum thermodynamic predictions in the future. To tackle this question we define the information-theoretic “projection” 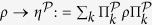 for a given initial quantum state *ρ* and a complete set of mutually orthogonal projectors 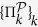. Such state transformation can be seen as analogous to the state transfer of erasure, 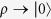, to a blank state 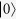. Physically, this projection can be interpreted as the result of an unread, or unselective^15^, measurement of an observable 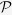 that has eigenvector projectors 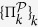. In an unselective measurement the individual measurement outcomes are not recorded and only the statistics of outcomes is known. In the literature the implementation of unselective measurements is often not specified, although it is typically thought of as measuring individual outcomes, e.g. with a Stern-Gerlach experiment, see Fig. 2a, followed by mixing. The crux is that the information-theoretic projection 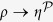 can be implemented in many physical ways. The associated thermodynamic heat and work will differ depending on *how* the projection was done and we will refer to the various realisations as “thermodynamic projection processes”. One possibility is decohering^16^ the state in the so-called pointer basis, 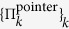, a thermodynamic process where an environment removes coherences in an uncontrolled manner resulting in no associated work. In general it is possible to implement the state transfer in a finely controlled fashion achieving optimal thermodynamic heat and work values.

Of particular importance in thermodynamics is the projection 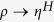 of the system’s initial state *ρ* onto the set of energy eigenstates 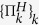 of the system’s Hamiltonian 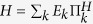 with *E*_*k*_ the energy eigenvalues. Here the state’s off-diagonals with respect to the energy eigenbasis are removed - a state transformation that is frequently employed in quantum thermodynamic derivations and referred to as “dephasing” or “measuring the energy”. Our key observation is that there exists a thermodynamic projection process realising this transformation and allowing to draw from the quantum system a non-trivial *optimal average work* of


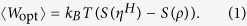


**Figure 1:**
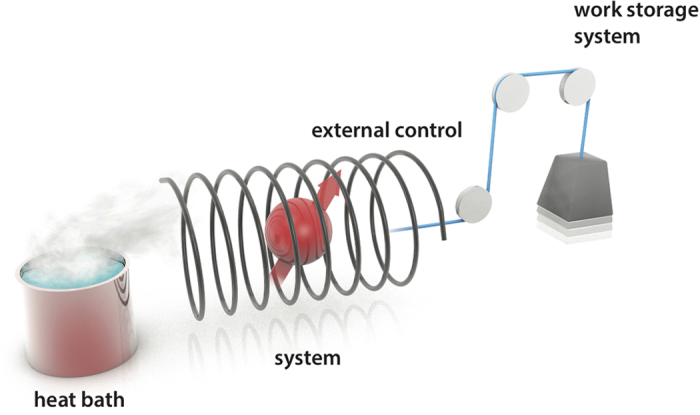
Thermodynamic setting. A system, depicted as a spin, interacts with a heat bath at temperature *T*, with which it exchanges *heat*, and with controlled energy sources, illustrated as coil and weight, with which it exchanges *work*. Work drawn from the system can be collected in a work storage system (weight) for future use.

Here *T* is the temperature of the heat bath with which the system is allowed to interact, see illustration Fig. 1, *k*_*B*_ is the Boltzmann constant and *S* is the von Neumann entropy. Crucially, this work is strictly positive for quantum states with coherences. Extending the key observation to general projections 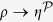 one finds that optimal thermodynamic projection processes can be implemented that allow to draw an average work of


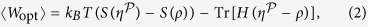


where an additional internal energy change term appears.

### Physical interpretation and assumptions made to derive the optimal work

The optimal work values stated in Eqs. (1) and (2) are valid for processes applied to classical and quantum states alike. While for a classical ensemble the entropy change, 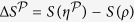, will be zero this is not so in the general quantum situation, where initial non-diagonal quantum states result in a strictly positive entropy change^17^. We note that while the optimal work values are in principle attainable, practical implementations may be suboptimal resulting in a reduced work gain or a higher work cost.

The physical meaning of 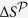 can be grasped by considering a lower bound^18^ on it, 
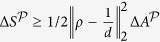
, see Supplement. Here *d* is the dimension of the system and 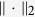 denotes the Hilbert-Schmidt norm. The first factor quantifies the distance of the initial state from the fully mixed state, while the second factor, 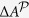, quantifies the angle between the diagonal basis of *ρ* and the projection basis 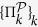. These terms correspond to incoherent and coherent mixing contributions. The entropy change is non-trivially bounded only if the initial state is not an incoherent mixture with respect to that basis. The entropy bound is the largest for pure initial states whose basis is mutually unbiased with respect to 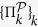. In this case the optimal entropy change is 
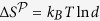
.

One may wonder where the work has gone to. There are two equivalent approaches to the accounting of work. In the present analysis the focus is on the work that the system exchanges, as done in statistical physics^5^,^19^,^20^,^21^,^22^. In this approach it is often not explicitly mentioned where the work goes to, but the only place work can go to are the externally controlled energy sources. Similarly, the heat, i.e. the energy change minus the work, is established implicitly. For example, in the experimental realisation of classical Landauer erasure with a colloidal silica bead trapped in an optical tweezer^21^, the dissipated heat of erasure was calculated by knowing the applied tilting forces and integrating over the bead’s dynamics. The second approach is to collect work in a separate work storage system^23^, as illustrated by the weight in Fig. 1 and detailed in the Supplement. Both the implicit and the explicit treatment of work are equivalent in the sense that the results obtained in one approach can be translated into the other.

The thermodynamic assumptions made to prove Eq. (2) are congruent with current literature^9^,^23^,^24^,^25^; specifically they are: (T0) an isolated system is a system that only exchanges work and not heat; (T1) the validity of the *first law* relating the internal energy change, Δ*U*, of the system during a process to its average heat absorbed and work drawn, 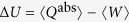; (T2) the validity of the *second law* relating the system’s entropy change to its average absorbed heat, 
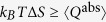
, when interacting with a bath at temperature *T*, with equality attainable by an optimal process; (T3) the thermodynamic entropy to be equal to the von Neumann entropy in equilibrium as well as out-of-equilibrium, 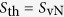. In addition we make the following standard quantum mechanics assumptions: (Q0) an isolated system evolves unitarily; (Q1) control of a quantum system includes its coherences. Details of the proof are in the Methods Summary. We note that in the single-shot setting whole families of second laws apply^7^,^8^ that differ from (T2) stated above. However, in the limit of infinitely many independent and identically prepared copies of the system these collapse to the standard second law, (T2), on the basis of which Eq. (2) is derived.

From the information-theory point of view the projections considered here constitute just one example of the larger class of trace-preserving completely positive (TPCP) maps characterising quantum dynamics. Of course, all TPCP maps can be interpreted thermodynamically with the assumptions stated above, resulting in an optimal average work given by a free energy difference. Erasure is another such map whose study forged the link between information theory and thermodynamics. The benefit of discussing “projections” here lies in the insight that this focus provides: it uncovers that coherences offer the potential to draw work making it a genuine and testable quantum thermodynamic feature. This work is non-trivial even when the thermodynamic process is operated on the system alone, not involving any side-information^14^ stored in other degrees of freedom.

### Qubit example for drawing optimal work

**Figure 2:**
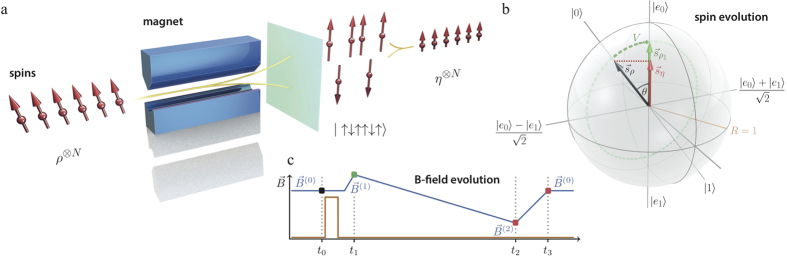
Two physical realisations of a projection process. (**a**) *N* identically prepared spin 1/2 particles in state 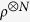 pass a Stern-Gerlach magnet and a screen after which they emerge in either the spin-up or the spin-down beam. Recombining the two beams mixes the spins to the final state 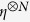 for *N* → ∞. Illustration of the spin example discussed in main text, showing the state evolution in (**b**) and the B-field evolution in (**c**). The poles in the Blochsphere (**b**) are the energy eigenstates 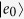 and 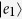 that are aligned and anti-aligned with an externally applied B-field (indicated in blue in (**c**)), which initially is 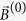 (black point in (**c**)). In the first step the Blochvector 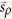 (black arrow in (**b**)) of Emmy’s initial state *ρ* is rotated on the green-dashed circle to 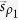 (green arrow in (**b**)). The unitary rotation *V* required for this step can be realised by applying a microwave pulse creating an additional B-field (indicated in orange in (**c**)) in the direction orthogonal to the plane of the green circle. At the end of the first step the pulse is turned off and the external B-field is adjusted to 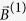 (green point in (**c**)). The second step shortens 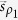 to 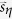 (red arrow in (**b**)), the Blochvector of *η* (superscripts *H* have been omitted). The external B-field (blue in (**c**)) decreases slowly to 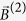 (red point at *t*_2_ in (**c**)). In the last step the B-field quickly returns to its initial value, 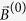 (red point at *t*_3_ in (**c**)), while the state remains *η*. The angle between the Blochvectors of *ρ* and *η* is indicated by *θ*.

To gain a detailed understanding of thermodynamic projection processes that give the optimal work stated in Eq. (1) we now detail one such process for the example of a spin-1/2 particle (qubit), see illustration in Fig. 2b,c. This process consists of a unitary evolution, a quasi-static evolution and a quench^25^, and it is optimal for any finite dimensional quantum system (proof in the Methods Summary). An experimentalist, Emmy, prepares the spin in a state 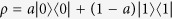 (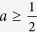 w.l.o.g.) exposed to an external magnetic field 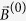 which she controls. The Hamiltonian associated with the system is 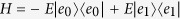 where the energy difference between the aligned ground state, 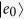, and anti-aligned excited state, 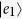, is given by 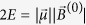 with 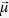 the spin’s magnetic moment. Importantly, in general the spin state’s basis, 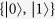, are superpositions with respect to the energy eigenbasis, 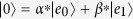 and 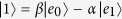 with 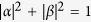. For the optimal implementation of the projection 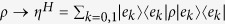 Emmy now proceeds with the following three steps.

Firstly, she isolates the spin from the bath and modifies external magnetic fields to induce a unitary rotation, 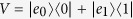, of the spin into the energy basis. In nuclear magnetic resonance (NMR)^26^ and pulsed electron spin resonance (ESR) experiments^27^ such rotations are routinely realised by radio-frequency and microwave pulses respectively, as evidenced by Rabi oscillations. The power, duration and phase of such a pulse would be chosen to generate the spin-rotation along the green circle until the desired unitary *V* is achieved. In the same step Emmy adjusts the strength of the external B-field such that the spin state 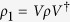 is Boltzmann-distributed at temperature *T* with respect to the energy gap of the Hamiltonian at the end of the step, *H*^(1)^. In NMR or ESR the B-field magnitude is tuned quickly on the *T*_1_ timescale to achieve the desired energy gap. In the second step, Emmy wants to implement a quasi-static evolution of the spin that is now thermal. She brings the spin in contact with the heat bath at temperature *T* and quasi-statically adjusts the magnitude of the external B-field allowing the spin state to thermalise at all times. The final B-field, 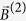, is chosen such that the final thermal state becomes *η*^*H*^. In ESR this step can be realised by changing the external B-field slowly on the *T*_1_ timescale so that the spin continuously equilibrates with its environment. Finally, Emmy isolates the spin from the environment and quickly changes the B-field to its original magnitude while the state remains *η*^*H*^.

During Step 1 and 3 the system was isolated and the average work drawn is thus just the average energy change. During Step 2 the average work is the equilibrium free energy difference between the final and initial thermal states at temperature *T*, see Supplement for details. In NMR/ESR the work contributions drawn from the spin system are done on the external B-field and the microwave mode. This could be detected by measuring the stimulated emission of photons in the microwave mode or observing current changes induced by the spins dynamics^26^,^27^. The overall thermodynamic process has now brought the spin from a quantum state with coherences, *ρ*, into a state without coherences, *η*^*H*^, while keeping the average energy of the spin constant. The net work drawn during the three steps adds up to 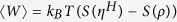 showing the attainability of the optimum stated in Eq. (1) for the spin-1/2 example. We note that Eq. (1) is also the maximal work that can be extracted from a *qubit* state *ρ* under *any* transformation of the system that conserves its average energy, 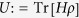, i.e. for qubits *η*^*H*^ is the optimal final state under this condition.

We emphasise that this optimal implementation involves a finely tuned and controlled operation that relies on knowledge of the initial state *ρ*. This is akin to the situation considered in^14^ where knowledge of the initial global state of system and memory is required for optimal erasure with side-information. It is important to distinguish this situation from that of Maxwell demon’s who has access to knowledge of the individual micro-states 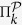 that make up the ensemble state 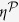, and who uses it to beat the second law^28^. In the scenario considered here there is no knowledge of the individual micro-states 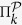 and the process does not violate the second law, on the contrary, it is derived from it.

### Comparison with single-shot work

The preceding discussion concerned the *average* work that can be drawn when operating on an ensemble of *N* → ∞ independent spins. This scenario contrasts with the single shot situation considered in a number of recent publications^7^,^14^,^29^,^30^. In particular, two major frameworks^29^,^30^ have recently been put forward to identify optimal *single-shot* work extraction and work cost of formation in the quantum setting. These frameworks rely on a resource theory approach^6^ and make use of min- and max-relative entropies that originate from one-shot information theory. The optimal work extraction schemes of these frameworks require non-diagonal states to be decohered first to become diagonal in the energy basis. This decoherence step is assumed to not have an associated single-shot work. However, the present analysis of energy basis projections showed that thermodynamic projection processes can yield positive average work, see Eq. (1). Therefore one may expect a positive work for removing coherences from a state *ρ* in the single-shot setting, too. Since our focus is the *N* → ∞ limit we will not aim to construct the single-shot case. Nevertheless, to establish a notion of consistency between single-shot results^29^,^30^ and the average analysis presented here we now separate the projection into a diagonal part that can be analysed in the single-shot framework and a non-diagonal part that can be analysed in the average framework. One possible decomposition of 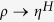 is the split in three steps each starting and ending with Hamiltonian *H*: 
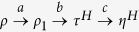
. Here *ρ*_1_ is the rotated state defined above and 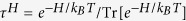 is the thermal state for the Hamiltonian *H* at temperature *T*. We can now use a single-shot analysis^30^ for Steps *b* and *c* that involve only states diagonal in the energy basis, giving a single-shot work contribution of 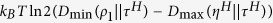, see Supplement. Here *D*_min_ and *D*_max_ are the min- and max-relative quantum entropies, respectively. Taking the limit of *N* → ∞ copies for Steps *b* and *c* and adding the average work contribution for the initial non-diagonal rotation *a*, 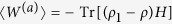, one indeed recovers the optimal average work as stated in Eq. (1). After making public our results very recently a paper appeared^31^ that derives the work that can be extracted when removing coherences in a single-shot setting. These results are in agreement with Eq. (1) and reinforce the above conclusion that coherences are a fundamental feature distinguishing quantum from classical thermodynamics.

### Comparison with quantum work fluctuation relations

**Figure 3:**
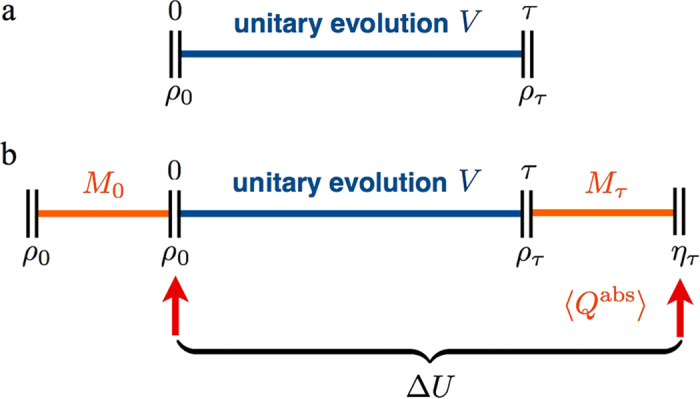
Dynamical steps in a quantum fluctuation experiment. (**a**) The quantum Jarzynski relation is described as characterising the non-equilibrium work of processes that start in a thermal state *ρ*_0_ and evolve unitarily (*V*), driven by a changing Hamiltonian, reaching the final state *ρ*_*τ*_ at time *τ*. This unitary process has no heat contribution. (**b**) Illustration of three steps that are assumed in mathematical derivations of the quantum Jarzynski relation^4^,^5^: initial energy measurement of *H*^(0)^ indicated by *M*_0_, unitary evolution, and final energy measurement of *H*^(*τ*)^ indicated by 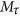. The ensemble state evolves here from *ρ*_0_ to *ρ*_*τ*_ and then to *η*_*τ*_, the state *ρ*_*τ*_ with its coherences removed. The observed average energy difference 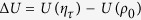 encompasses both, the unitary process and the second projection process, and can in general contain a heat contribution 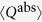, in contrast to (**a**).

The key observation was that thermodynamic projection processes can have a non-trivial work and heat. Another instance where this has interesting repercussions is the quantum Jarzynski equality^4^,^5^. This is a generalisation of the prominent classical fluctuation relation valid for general non-equilibrium processes, which has been used to measure the equilibrium free energy surface inside bio-molecules by performing non-equilibrium pulling experiments^19^. The quantum version has recently been tested for the first time in a nuclear magnetic resonance experiment^26^. The quantum Jarzynski relation, 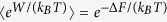, links the fluctuating work, *W*, drawn from a system in individual runs of the same non-equilibrium process, with the free energy difference, Δ*F*, of the thermal states of the final and initial Hamiltonian, see Supplement. In its derivation a system initially in a thermal state *ρ*_0_ with respect to Hamiltonian *H*^(0)^ at temperature *T* is first measured in the energy basis of *H*^(0)^. The Hamiltonian is then varied in time ending in *H*^(*τ*)^ generating a unitary evolution, *V*, of the system, see Fig. 3a. A second measurement, in the energy basis of *H*^(*τ*)^, is then performed to establish the final fluctuating energy. For each run the difference of the two measured energies has been associated with the fluctuating work^5^, Δ*E* = −*W*. The experiment is repeated, each time producing a fluctuating work value. On average the work extracted from the system during the quantum non-equilibrium process turns out to be 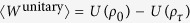 where 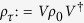 is the ensemble’s state after the unitary evolution, and similarly the average exponentiated work is calculated. The above identification 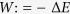 was made assuming that the system undergoes a unitary process with no heat dissipation. However, the need to acquire knowledge of the system’s final energies requires the second measurement. The ensemble state is thus further altered from *ρ*_*τ*_ to *η*_*τ*_, the state *ρ*_*τ*_ with any coherences in the energy basis of *H*^(*τ*)^ removed. This step is not unitary - during the projection 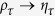 the system may absorb heat, 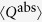, indicated in Fig. 3b, whose value depends on *how* the process is conducted. Thus, while the energy difference for the projection is zero, 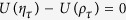, for states *ρ*_*τ*_ with coherences the entropy difference is not trivial, 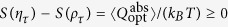. This implies that in an experimental implementation of the Jarzynski relation the work done by the system on average can be more than previously thought, 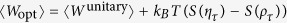. We conclude that the suitability of identifying 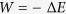, and hence the validity of the quantum Jarzynski *work* relation, depends on the details of the physical process that implements the second measurement. This conclusion is not at odds with previous experiments^26^ which showed nature’s agreement with 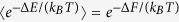, involving the average of the exponentiated measured fluctuating energy.

### Work from coherences of correlated quantum systems

It is insightful to extend the thermodynamic analysis of projections to correlated systems. An experimenter may have access not only to the system *S* but also the auxiliary systems *A* with which *S* is correlated^14^. She can then perform a global operation, 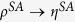, that implements a projection 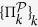 locally on the system *S*, i.e. 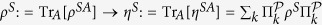, while leaving the reduced state of the auxiliary system unchanged, i.e. 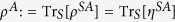. By doing so the experimenter can optimally draw the overall work 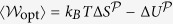, where 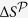 is the entropy change for the state of system + auxiliary and 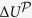 is still the energy change of the system *alone*. This quantity can be re-written as the sum of two terms: 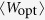, the extractable work when operating on the system alone given in Eq. (2), and 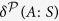, a positive term quantifying the quantum correlations between *S* and *A*, see Supplement. The latter contribution was previously identified in an inspiring paper by Zurek^13^. It depends on the choice of projectors and is related to, but broader than, quantum discord^32^ which is optimised over all possible projectors. This means that even states of system and auxiliary that can be considered classically correlated (i.e. no discord) provide an advantage for drawing work contrasting with the erasure process where this only occurs for highly entangled states^14^. The gap between these two sets of correlated states is an intriguing fact and calls for further exploration of the link between thermodynamics and information theory in the quantum regime.

## Discussion of implications

To conclude, erasure is not the only irreversible information processing task – in the quantum regime a second fundamental process exists that mirrors Landauer’s erasure. In contrast to the minimum heat limit of erasure, thermodynamic projection processes have a maximum work limit. While the former is non-zero for the erasure of classical *and* quantum bits, optimal thermodynamic projection processes have a non-zero work *only* when applied to quantum states with coherences. The optimal average work stated in Eqs. (1) and (2) constitutes an experimentally accessible quantum thermodynamic prediction. Future experiments testing this optimal work may be pursued with current setups, for instance with NMR/ESR techniques^26^,^27^ or single atoms^33^, and promise to be accessible with other platforms entering the quantum regime, such as single electron boxes^22^. Experiments will be limited by practical constraints, such as achieving a quasistatic process and obtaining the maximum work for pure states which may require, for instance, very large B-fields.

The derivation of the optimal work value is mathematically straightforward, just like that of Landauer’s principle. The result’s significance is that it opens new avenues of thought and provides key input for the construction of a future quantum thermodynamic framework. For example, the developed approach opens the door to investigate the connection between microscopic statistical physics and macroscopic thermodynamics in the quantum regime. While it is straightforward to identify the thermodynamic work of quantum processes involving macroscopic ensembles, what is needed is a microscopic concept of work that when averaged, gives the correct macroscopic work. The microscopic work concept should be valid for general (open) quantum processes and quantum states (including coherences), and only require access to properties of the system. While single-shot approaches have discarded coherences^29^,^30^, fluctuating work approaches cannot be applied directly to a system undergoing open quantum evolution^20^.

The observation is also important from the experimental perspective as testing quantum thermodynamic predictions will involve measurement – a projection process. We have argued that measurements, such as those required in establishing the Jarzynski equality, are not necessarily thermodynamically neutral. Indeed, they can be implemented in different physical ways and in general play an active role in thermodynamics, contributing a non-zero average heat and work. This new perspective gives physical meaning to the change of entropy in the debated quantum measurement process - it provides a capacity to draw work. Specifically, work can be drawn when *coherences* of a state are removed during an unselective measurement.

Finally, it is apparent that optimal thermodynamic projection processes require use of knowledge of the initial state *ρ*, i.e. its basis and eigenvalues. One may be inclined to exclude use of such knowledge, particularly when considering projections in the context of measurement which is often associated with the acquisition of knowledge. Such restriction would necessarily affect the set of assumptions (T0-T3, Q0-Q1) in the quantum regime. These could be changed, for example, by dropping the possibility of saturating the second law inequality (cf. T2) or choosing a new quantum non-equilibrium entropy that only considers the state’s diagonal entries (cf. T3). The latter would mean a departure from standard quantum information theory where entropies are basis-independent. Thus whichever approach one takes - not making or making a restriction - quantum coherences will contribute a new dimension to thermodynamics. They either lead to non-classical work extraction or they alter the link between information theory and thermodynamics in the quantum regime. The line drawn here between the assumptions (T0-T3, Q0-Q1) and results (Eqs. (1) and (2)) establishes a frame for this possibility to be investigated.

## Methods Summary

Further underlying research materials can be accessed in the supplementary information that accompanies this article.

### Proof of Eq. (2)

Using the first law (T1) the average work drawn in a thermodynamic projection process 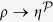 is simply 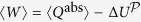, where 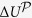 is the average energy change for that process. Relating the average heat absorbed by the system during the process to its entropy change one then obtains 
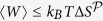
 (T2). Here 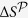 is the difference of von Neumann entropies of the system’s state before and after the projection (T3). The average work drawn is thus 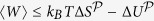, where the entropy change is non-negative and the energy change can be either positive or negative. The stated *optimal* work, 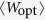, is achieved when the inequality is saturated by an optimal process (T2) the implementation of which may require knowledge of the initial state and control of coherences (Q1). In the special case of a projection onto the energy eigenbasis 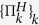 the internal energy change is zero, 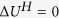, and one obtains Eq. (1).

### Optimality of three-step process for finite-dimensional systems

It is straightforward to generalise the proof of optimality from the two-dimensional spin-1/2 example to thermodynamic projection processes in dimension *d*. Again the projectors 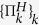 map onto the energy eigenspaces of the Hamiltonian, 
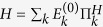
, where 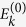, 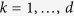, are the energy eigenvalues. A general initial state can be written as 
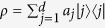
 where 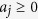 are probabilities, 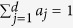, 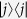 are rank-1 projectors on the corresponding eigenvectors 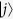, and 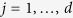. A unitary operation, *V*, is now chosen such that it brings the initial configuration (*ρ*, *H*) into the new diagonal and thermal configuration 
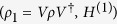
 where 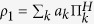 and 
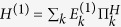
. The new energy eigenvalues, 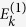, are adjusted such that the probabilities *a*_*k*_ are thermally distributed with respect to *H*^(1)^ for the bath temperature *T*. Adjusting the Hamiltonian eigenvalues while letting the state thermalise at all times now results in a isothermal quasi-static operation from 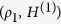 to 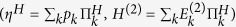. Here the new energy eigenvalues, 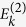, are chosen to be thermal (at *T*) for the state’s probabilities which are given by 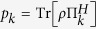. Finally, a quench brings the thermal configuration 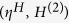 quickly into the non-equilibrium state 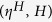. The average work for this overall process is 
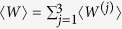
 where 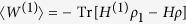 and 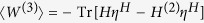 because the first and third steps are unitary (Q0 + T0). The quasistatic step’s work is^25^,^29^ 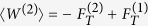 where 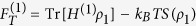 is the thermal equilibrium free energy for Hamiltonian *H*^(1)^, and similarly, 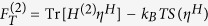. Summing up and using 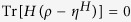, one obtains 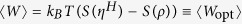 concluding the optimality proof of the process sequence.

## Additional Information

**How to cite this article**: Kammerlander, P. and Anders, J. Coherence and measurement in quantum thermodynamics. *Sci. Rep.* **6**, 22174; doi: 10.1038/srep22174 (2016).

## Supplementary Material

Supplementary Information

## References

[b1] Scully M. O., Zubairy M. S., Agarwal G. S. & Walther H. Extracting Work from a Single Heat Bath via Vanishing Quantum Coherence. Science 299, 862 (2003). 12511655 10.1126/science.1078955

[b2] Kosloff R. & Levy A. Quantum Heat Engines and Refrigerators: Continuous Devices. Annu. Rev. Phys. Chem. 65, 365 (2014). 24689798 10.1146/annurev-physchem-040513-103724

[b3] Roßnagel J., Abah O., Schmidt-Kaler F., Singer K. & Lutz E. Nanoscale Heat Engine Beyond the Carnot Limit. Phys. Rev. Lett. 112, 030602 (2014). 24484127 10.1103/PhysRevLett.112.030602

[b4] Mukamel S. Quantum extension of the Jarzynski relation: Analogy with stochastic dephasing. Phys. Rev. Lett. 90, 170604 (2003). 12786064 10.1103/PhysRevLett.90.170604

[b5] Talkner P., Lutz E. & Hänggi P. Fluctuation theorems: Work is not an observable. Phys. Rev. E 75, 050102 (R) (2007). 10.1103/PhysRevE.75.050102 17677006

[b6] Janzing D., Wocjan P., Zeier R., Geiss R. & Beth T. Thermodynamic cost of reliability and low temperatures: tightening Landauer’s principle and the second law. Int. J. Theor. Phys. 39, 2717 (2000).

[b7] Brandão F. G. S. L., Horodecki M., Ng N. H. Y., Oppenheim J. & Wehner S. The second laws of quantum thermodynamics. PNAS 112, 3275 (2015). 25675476 10.1073/pnas.1411728112 PMC4372001

[b8] Lostaglio M., Jennings D. & Rudolph T. Description of quantum coherence in thermodynamic processes requires constraints beyond free energy. Nat. Commun. 6, 6383 (2015). 25754774 10.1038/ncomms7383 PMC4366492

[b9] Landauer R. Dissipation and heat generation in the computing process. IBM J. Res. Develop. 5, 148–156 (1961).

[b10] Jacobs K. Quantum measurement and the first law of thermodynamics: The energy cost of measurement is the work value of the acquired information. Phys. Rev. E 86 040106(R) (2012). 10.1103/PhysRevE.86.040106 23214518

[b11] Erez N. Thermodynamics of projective quantum measurements. Phys. Scr. 151, 014028 (2012).

[b12] Åberg J. Catalytic Coherence. Phys. Rev. Lett. 113, 1504022 (2014). 10.1103/PhysRevLett.113.150402 25375693

[b13] Zurek W. H. Quantum discord and Maxwell’s demons. Phys. Rev. A 67, 012320 (2003).

[b14] del Rio L., Åberg J., Renner R., Dahlsten O. & Vedral V. The thermodynamic meaning of negative entropy. Nature 474, 61 (2011). 21637254 10.1038/nature10123

[b15] Erez N., Gordon G., Nest M. & Kurizki G. Thermodynamic control by frequent quantum measurements. Nature 452, 724 (2008). 18401404 10.1038/nature06873

[b16] Zurek W. H. Decoherence, einselection, and the quantum origins of the classical. Rev. Mod. Phys. 75, 715 (2003).

[b17] Nielsen M. A. & Chuang I. L. Quantum Computation and Quantum Information Cambridge University Press, Cambridge UK (2000).

[b18] Streater R. F. Convergence of the Quantum Boltzmann Map. Commun. Math. Phys. 98, 177 (1985).

[b19] Liphardt J., Dumont S., Smith S. B., Tinoco I. & Bustamante C. Equilibrium information from nonequilibrium measurements in an experimental test of Jarzynski’s equality. Science 296, 1832 (2002). 12052949 10.1126/science.1071152

[b20] Campisi M., Talkner P. & Hänggi P. Fluctuation theorem for arbitrary open quantum systems. Phys. Rev. Lett. 102, 210401 (2009). 19519085 10.1103/PhysRevLett.102.210401

[b21] Bérut A., Arakelyan A., Petrosyan A., Ciliberto S., Dillenschneider R. & Lutz E. Experimental verification of Landauer’s principle linking information and thermodynamics. Nature 483, 7388 (2012). 10.1038/nature10872 22398556

[b22] Saira O.-P. *et al.* Test of the Jarzynski and Crooks Fluctuation Relations in an Electronic System. Phys. Rev. Lett. 109, 180601 (2012). 23215263 10.1103/PhysRevLett.109.180601

[b23] Skrzypczyk P., Short A. J. & Popescu S. Work extraction and thermodynamics for individual quantum systems. Nat. Commun. 4, 4185 (2013). 10.1038/ncomms5185 24969511

[b24] Esposito M., Lindenberg K. & Van den Broeck Ch. Entropy production as correlation between system and reservoir. New J. Phys. 12, 013013 (2010).

[b25] Anders J. & Giovanetti V. Thermodynamics of discrete quantum processes. New J. Phys. 15, 033022 (2013).

[b26] Batalhao T. B. *et al.* Experimental Reconstruction of Work Distribution and Study of Fluctuation Relations in a Closed Quantum System. Phys. Rev. Lett. 113, 140601 (2014). 25325627 10.1103/PhysRevLett.113.140601

[b27] Morley G. W. *et al.* The initialization and manipulation of quantum information stored in silicon by bismuth dopants. Nat. Mat. 9, 725 (2010). 10.1038/nmat2828 20711180

[b28] Maruyama K., Nori F. & Vedral V. Colloquium: The physics of Maxwell’s demon and information. Rev. Mod. Phys. 81, 1 (2009).

[b29] Åberg J. Truly work-like work extraction via a single-shot analysis. Nat. Commun. 4, 1925 (2013). 23801350 10.1038/ncomms2712

[b30] Horodecki M. & Oppenheim J. Fundamental limitations for quantum and nanoscale thermodynamics. Nat. Commun. 4, 2059 (2013). 23800725 10.1038/ncomms3059

[b31] Korzekwa K., Lostaglio M., Oppenheim J. & Jennings D. The extraction of work from quantum coherence. arXiv:1506.07875 (2015).

[b32] Henderson L. & Vedral V. Classical, quantum and total correlations. J. Phys. A: Math. Gen. 34, 6899 (2001).

[b33] Maunz P. Gentle measurement. Nature 475, 180 (2011); Volz, J., Gehr, R., Dubois G., Estève, J. & Reichel, J. Measurement of the internal state of a single atom without energy exchange. *Nature* **475,** 210 (2011). 21753851 10.1038/nature10225

