# Supplementary material for: Coherence and measurement in quantum thermodynamics
Source: Sci Rep. 2016 Feb 26;6:22174. doi: 10.1038/srep22174 (PMC4768176; doi:10.1038/srep22174)
Supplement: Supplementary Information [file srep22174-s1.pdf]

# Supplementary Information

## Coherence and measurement in quantum thermodynamics

**P Kammerlander* and J Anders†**

## A Spin Example

We here detail the three steps of the optimal thermodynamic projection process of the spin system discussed in the main text. Emmy starts with a spin in state

$$\rho = a\,|0\rangle\langle 0| + (1-a)\,|1\rangle\langle 1|, \tag{SI.1}$$

with Blochvector

$$\vec{s}_\rho := \mathrm{Tr}[\rho\,\vec{\sigma}] = (2a-1)\,\hat{0}, \tag{SI.2}$$

where $\vec{\sigma}$ is the vector of the three Pauli matrices, $\sigma_1, \sigma_2$ and $\sigma_3$ and $\hat{0} = \mathrm{Tr}[|0\rangle\langle 0|\,\vec{\sigma}]$ is the unit vector in the Blochsphere pointing from the origin to the state $|0\rangle$, see Fig. SI.1a. We assume without loss of generality that $a \geq \frac{1}{2}$. If this was not the case, the labels $|0\rangle\langle 0|$ and $|1\rangle\langle 1|$ should be interchanged. The spin's initial Hamiltonian is given by $H = -E\left(\Pi_0^H - \Pi_1^H\right)$, where $\Pi_k^H = |e_k\rangle\langle e_k|$ with $k = 0, 1$ are the rank-1 projectors onto the two energy eigenstates and $E > 0$. This Hamiltonian arises when the spin is exposed to an external magnetic field $\vec{B}^{(0)}$. The energy separation of the aligned ground state, $|e_0\rangle$, and anti-aligned excited state, $|e_1\rangle$, is $2E = 2\,|\vec{\mu}|\,|\vec{B}^{(0)}|$, where $\vec{\mu}$ is the magnetic moment of the spin. A general initial state $\rho$ is not diagonal in the basis $\{|e_0\rangle, |e_1\rangle\}$, in other words the spin's eigenstates are superpositions with respect to the energy eigenbasis, $|0\rangle = \alpha^*\,|e_0\rangle + \beta^*\,|e_1\rangle$ and $|1\rangle = \beta\,|e_0\rangle - \alpha\,|e_1\rangle$ with $|\alpha|^2 + |\beta|^2 = 1$. The spin's Blochvector, $\hat{0}$, is then *not* parallel to the B-field, $\vec{B}^{(0)}$. Emmy wants to obtain the state where the coherences with respect to the energy basis $\{|e_0\rangle, |e_1\rangle\}$ have been removed,

$$\eta^H \;= \textstyle\sum_{k=0,1} \Pi_k^H\,\rho\,\Pi_k^H = \sum_{k=0,1} \mathrm{Tr}[\Pi_k^H\,\rho]\,\Pi_k^H = p\;\Pi_0^H + (1-p)\;\Pi_1^H, \tag{SI.3}$$

where $p = \mathrm{Tr}[\Pi_0^H\,\rho]$ is the probability for obtaining outcome $|e_0\rangle$ in a measurement of $H$. Here $\eta^H$ has the same average energy as the initial state $\rho$,

$$\mathrm{Tr}[\eta^H\,H] = \sum_{k=0,1} \mathrm{Tr}[\,\Pi_k^H\,\rho\,\Pi_k^H\,H\,] = \sum_{k=0,1} \mathrm{Tr}[\,\rho\,\Pi_k^H\,H\,\Pi_k^H\,] = \mathrm{Tr}[\rho\,H], \tag{SI.4}$$

where we used orthonormality and completeness of the projectors $\{\Pi_0^H, \Pi_1^H\}$. The Blochvector of the final state is defined as

$$\vec{s}_\eta := \mathrm{Tr}[\eta^H\,\vec{\sigma}] = (2p-1)\,\hat{e}_0 \tag{SI.5}$$

where $\hat{e}_0 = \mathrm{Tr}[|e_0\rangle\langle e_0|\,\vec{\sigma}]$ is the unit vector in the Blochsphere pointing from the origin to the state $|e_0\rangle$. Since geometrically the mapping $\rho \to \eta^H$ is a projection of $\vec{s}_\rho$ onto the vertical axis in the Blochsphere, the length of the final Blochvector, $\vec{s}_\eta$, is shorter than the initial Blochvector, $\vec{s}_\rho$. This shortening is associated with an entropy increase[1]. When describing the process in the following we assume that $p \geq \frac{1}{2}$ in accordance with the illustration in Fig. SI.1a. At the end of this section we come back to the case $p < \frac{1}{2}$.

Emmy proceeds with three steps made up of quantum thermodynamic primitives with known work and heat contributions[2], Fig. SI.1a & SI.1b:

$$(\rho, H) \stackrel{1}{\longrightarrow} (\rho_1, H^{(1)}) \stackrel{2}{\longrightarrow} (\eta^H, H^{(2)}) \stackrel{3}{\longrightarrow} (\eta^H, H). \tag{SI.6}$$

In the first step, $(\rho, H) \stackrel{1}{\longrightarrow} (\rho_1, H^{(1)})$, Emmy isolates the spin from the bath and rotates the B-field such that the variation of the field induces a unitary transformation of the spin into the energy eigenbasis, with unitary $V = |e_0\rangle\langle 0| + |e_1\rangle\langle 1|$. The state after this step is

$$\rho_1 = V\,\rho\,V^\dagger = a\;\Pi_0^H + (1-a)\;\Pi_1^H. \tag{SI.7}$$

* Institute for Theoretical Physics, ETH Zurich, 8093 Zurich, Switzerland.
† Physics & Astronomy, University of Exeter, Exeter EX4 4QL, UK.

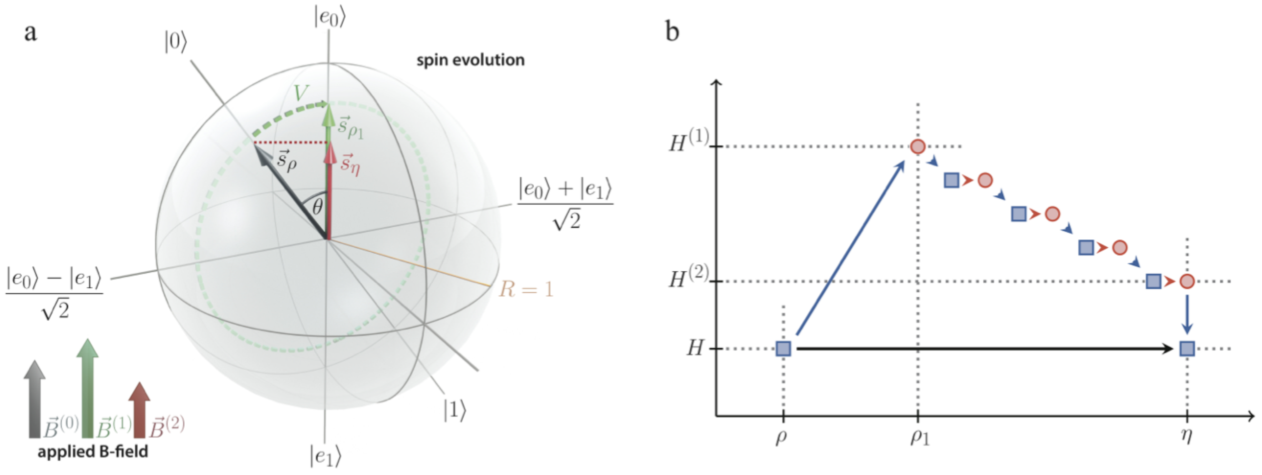


**Figure SI.1: Illustration of optimal three step process in the Blochsphere (a) and in configuration space (b).** For readability the superscript of $\eta$ has been dropped. **a,** The Blochvector of a spin-1/2 state, $\vec{s}_\rho$, is shown as the black arrow in the sphere, and it is exposed to an external B-field, $\vec{B}^{(0)}$, indicated on the left. The first step rotates $\vec{s}_\rho$ on the green-dashed circle to the green arrow $\vec{s}_{\rho_1}$, while the B-field changes to $\vec{B}^{(1)}$. The second step shortens $\vec{s}_{\rho_1}$ to $\vec{s}_\eta$ while the B-field decreases to $\vec{B}^{(2)}$. In the last step the B-field returns to its initial value, $\vec{B}^{(0)}$, while the state remains $\eta^H$. **b,** Thermodynamic steps can be illustrated in the configuration space of pairs of states and Hamiltonians[2]. Unitary evolutions are shown as blue arrows while thermalization processes are indicated by red horizontal arrows. Thermal states are denoted by red circles and non-equilibrium configurations by blue squares. The three step process is also optimal for any finite-dimensional quantum system (see Methods Summary). It starts with a unitary transforming the initial non-equilibrium configuration $(\rho, H)$ to the thermal configuration $(\rho_1, H^{(1)})$. A quasi-static process then brings $(\rho_1, H^{(1)})$ to $(\eta^H, H^{(2)})$, illustrated as infinitesimally small steps consisting of unitary evolution followed by thermalization. Finally, a unitary quench from the thermal configuration $(\eta^H, H^{(2)})$ to the non-equilibrium configuration $(\eta^H, H)$ concludes the process.

The B-field after this step, $\vec{B}^{(1)}$, is chosen such that the new Hamiltonian $H^{(1)} = -E^{(1)}\left(\Pi_0^H - \Pi_1^H\right)$ has eigenvalues $E^{(1)} = |\vec{\mu}|\,|\vec{B}^{(1)}| = \frac{k_B T}{2}\ln\frac{a}{1-a}$, where $k_B$ is the Boltzmann constant and $T$ is the temperature of the heat bath that Emmy will use in the next step. This choice of the B-field makes the state $\rho_1$ a thermal state with respect to $H^{(1)}$ at temperature $T$, i.e. $\rho_1 = \frac{e^{-\beta H^{(1)}}}{Z^{(1)}}$ with $Z^{(1)} = \mathrm{Tr}\left[e^{-\beta H^{(1)}}\right]$ and inverse temperature $\beta = \frac{1}{k_B T}$. Since the system was isolated in the first step no heat exchange was possible and the entire average energy change of the system is drawn from the system as work $\langle W^{(1)}\rangle = -\mathrm{Tr}[\rho_1 H^{(1)} - \rho H]$. Physical constraints may make this process difficult to realise, for instance, pure initial states would require a B-field, $B^{(1)}$, of infinite magnitude because thermal states at any finite temperature are only pure if the energy gap is infinite. In this case there is a trade-off between the maximal magnitude the B-field can reach and the precision with which the process is carried out. In the following we assume that the maximal B-field is large enough to make the error in the precision negligibly small.

In the second step, $(\rho_1, H^{(1)}) \xrightarrow{2} (\eta^H, H^{(2)})$, Emmy brings the spin in contact with the bath at temperature $T$, not affecting the spin's state as it is already thermal. She then quasi-statically decreases the magnitude of the B-field, while keeping the system in contact with the bath at all times, such that the final Hamiltonian is $H^{(2)} = -E^{(2)}\left(\Pi_0^H - \Pi_1^H\right)$ where the B-field is chosen such that $E^{(2)} = \frac{k_B T}{2}\ln\frac{p}{1-p}$ where $p$ is the probability of measuring $-E$ in the initial state, $\rho$. The quasi-static evolution means that the system is thermalised at all times, arriving in the final state

$$\frac{e^{-\beta H^{(2)}}}{Z^{(2)}} = p\,\Pi_0^H + (1-p)\,\Pi_1^H = \eta^H \tag{SI.8}$$

which is thermal with respect to $H^{(2)}$ where $Z^{(2)} = \mathrm{Tr}\left[e^{-\beta H^{(2)}}\right]$. This state is exactly $\eta^H$, the desired final state after the projection. The quasi-static process considered here has a known average work given by the free energy difference[2–4], $\langle W^{(2)}\rangle = -\left(F_T^{(2)}(\eta^H) - F_T^{(1)}(\rho_1)\right)$ where $F_T^{(2)}(\eta^H) = \mathrm{Tr}[\eta^H H^{(2)}] - k_B T\, S(\eta^H)$ and $F_T^{(1)}(\rho_1) = \mathrm{Tr}[\rho_1 H^{(1)}] - k_B T\, S(\rho_1)$ are standard thermal equilibrium free energies and $S$ is the von Neumann entropy defined by $S(\eta^H) = -\mathrm{Tr}[\eta^H \log \eta^H]$ and likewise for $\rho_1$.

Finally, in the third step, $(\eta^H, H^{(2)}) \xrightarrow{3} (\eta^H, H)$, Emmy isolates the spin from the bath and changes the energy levels of the Hamiltonian such that it becomes the initial Hamiltonian $H$ again. This step is done quickly so that the state of the spin does not change. Because the system is isolated the energy change in this step is entirely due to work $\langle W^{(3)}\rangle = -\mathrm{Tr}[\eta^H (H - H^{(2)})]$. In total, this thermodynamic process has brought the spin

from the quantum state $(\rho, H)$ to the state $(\eta^H, H)$ while not changing the energy of the spin, $\mathrm{Tr}[(\rho-\eta^H)\, H] = 0$. The overall average work drawn from the spin is

$$\begin{aligned}\langle W\rangle &= \langle W^{(1)}\rangle + \langle W^{(2)}\rangle + \langle W^{(3)}\rangle \\ &= -\mathrm{Tr}[\rho_1\, H^{(1)}] + \mathrm{Tr}[\rho\, H] - \mathrm{Tr}[\eta^H\, H^{(2)}] + k_B T\, S(\eta^H) + \mathrm{Tr}[\rho_1\, H^{(1)}] - k_B T\, S(\rho_1) - \mathrm{Tr}[\eta^H\, (H - H^{(2)})] \\ &= k_B T\, (S(\eta^H) - S(\rho_1)) \\ &= k_B T\, (S(\eta^H) - S(\rho)) \equiv \langle W_{\mathrm{opt}}\rangle,\end{aligned} \tag{SI.9}$$

showing the optimality of the three step process for the spin example, cf. Eq. (1) in the main text.

The above example assumed $p \geq \frac{1}{2}$. Suppose now that the probability to find the final state $\eta^H$ in the ground state $|e_0\rangle$ with respect to the Hamiltonian $H$ was smaller than to find it in the excited state $|e_1\rangle$, i.e. $p < \frac{1}{2}$. Proceeding through the three steps described one finds that the mathematics is exactly the same. In particular, after Step 2 $\eta^H$ is a thermal state with respect to $H^{(2)}$ at inverse temperature $\beta$. The only difference occurs in the interpretation as for the Hamiltonian $H^{(2)}$ the ground state is $|e_1\rangle$ because $E^{(2)} = \frac{k_B T}{2} \ln \frac{p}{1-p} < 0$ is negative. This is feasible by making the B-field $B^{(2)}$ negative, thus swapping the ground and the excited state. Consequently the analysis above and the resulting expression of the total extracted work remain the same.

The work extracted in the individual steps of the thermodynamic projection process can be either positive or negative, depending on the initial state $\rho$, the Hamiltonian $H$ and the temperature $T$ of the heat bath. Their sum, $\langle W\rangle$, is strictly positive whenever the initial state was not diagonal in the energy eigenbasis, a consequence of the entropy increase[1] from $\rho$ to $\eta^H$. On the other hand for classical states – all diagonal in the energy basis – the optimal work for such a projection is always zero. The Methods Summary in the main text extends the optimality proof of the above three step process, illustrated in Fig SI.1b, to the general finite-dimensional case.

**A note on optimal work extraction at constant average energy.** Assume we are given an initial state $\rho$ and a non-degenerate Hamiltonian $H$ for a quantum system. The goal is to find the maximal work that can be obtained in a thermodynamic process that involves a heat bath at temperature $T$ under the restriction that the average energy of the system after the process is the same as it was before the process, $U := \mathrm{Tr}[\rho\, H]$. Using Eq. (2) in the main text together with the condition that internal energy does not change this amounts to finding the maximum over the set of states $\sigma$ with $\mathrm{Tr}[\sigma\, H] = U$,

$$\langle W^{\max}\rangle := \max_{\sigma} k_B\, T(S(\sigma) - S(\rho)) = k_B\, T(\max_{\sigma} S(\sigma) - S(\rho)). \tag{SI.10}$$

It is well-known that at a fixed expectation value of an observable $H$ the Gibbs states $\sigma_\lambda = e^{-\lambda H}/\mathrm{Tr}[e^{-\lambda H}]$ are the states of maximal entropy[13,14]. Here the parameter $\lambda$ has to be chosen such that the energy of the Gibbs state matches $U$ - therefore there is only one $\sigma_{\lambda^*}$, with $\lambda^*$ such that $\mathrm{Tr}[\sigma_{\lambda^*}\, H] \equiv U$, that gives the maximum here. The maximum entropy is then

$$S(\sigma_{\lambda^*}) = -\mathrm{Tr}[\sigma_{\lambda^*} \ln \sigma_{\lambda^*}] = -\mathrm{Tr}[\sigma_{\lambda^*}(-\lambda^* H - \ln \mathrm{Tr}[e^{-\lambda^* H}])] = \lambda^*\, U + \ln \mathrm{Tr}[e^{-\lambda^* H}] \tag{SI.11}$$

and the maximum average work that can be extracted from $\rho$ at fixed average energy $U$ is then

$$\langle W^{\max}\rangle = k_B\, T(\lambda^*\, U + \ln \mathrm{Tr}[e^{-\lambda^* H}] - S(\rho)), \tag{SI.12}$$

For the special case that the system is a qubit (two-dimensional) the optimum Gibbs state for work extraction $\sigma_{\lambda^*}$ is identical to the projected state $\eta^H = \sum_{k=0,1} \Pi^{(k)}\, \rho\, \Pi^{(k)}$ and the maximal work that can be drawn from a system starting in state $\rho$, while keeping its average energy fixed, is $\langle W_{\mathrm{opt}}\rangle$ in Eq. (1) in the main text. To see this we expand $H = E^{(0)}\, \Pi^{(0)} + E^{(1)}\, \Pi^{(1)}$ and $U = p\, E^{(0)} + (1-p) E^{(1)}$ with $p := \mathrm{Tr}[\rho\, \Pi^{(0)}]$. Now here $\lambda^*$ must be chosen such that $\sigma_{\lambda^*} = e^{-\lambda^* H}/\mathrm{Tr}[e^{-\lambda^* H}] = p\, \Pi^{(0)} + (1-p)\, \Pi^{(1)}$, i.e. $p = e^{-\lambda^* E^{(0)}}/(e^{-\lambda^* E^{(0)}} + e^{-\lambda^* E^{(1)}})$, so that $\sigma_{\lambda^*}$ has just the right energy $\mathrm{Tr}[\sigma_{\lambda^*}\, H] = U$. On the other hand the projection state has the same expansion, $\eta^H = p\, \Pi^{(0)} + (1-p)\, \Pi^{(1)} = \sigma_{\lambda^*}$. We note that this coincidence is not true for higher dimensional systems where the energy-projected state $\eta^H$ will in general have a non-monotonous, non-canonical distribution in its energy eigenbasis, while $\sigma_{\lambda^*}$ must be Gibbs-distributed.

Considering the illustration in Fig. SI.1a, the qubit states $\sigma$ fulfilling the condition $\mathrm{Tr}[\sigma\, H]$ are located on the plane which contains $\rho$ and is perpendicular to the $|e_0\rangle$-$|e_1\rangle$-axis. On the other hand, in the Bloch picture a state has higher entropy the closer it is to the center of the sphere. Hence, the optimal final state when extracting work from $\rho$ while conserving the average energy of the system is the state $\rho$ projected to the $|e_0\rangle$-$|e_1\rangle$-axis, i.e. $\eta^H$.

## B Work storage system

In the previous section it was stated that work can be drawn from a quantum system when undergoing a thermodynamic projection process. But where has the work gone to?

There are two approaches of accounting for work that are mirror images to each other. One approach[2–10] focusses on the work that the system exchanges, as described above. Here it is often not explicitly mentioned where the work goes to, but the only place it can go to are the externally controlled energy sources, see Fig. 1 in the main text. Another way of accounting is to explicitly introduce a work system to store the work drawn[11,12]. One way of doing so in an average scenario is to introduce[11] a 'suspended weight on a string', described by a quantum system $W$, that could be raised or lowered to store work or draw work from it. Specifically, the Hamiltonian of the work storage system is defined as $H^W = m\, g\, x$, representing the energy of a weight of mass $m$ in the gravitational field with acceleration $g$ at height $x$. In addition, an explicit thermal bath $B$ is introduced[13,14] consisting of a separate quantum system in a thermal (or Gibbs) state $\tau^B$. Both, the explicit work storage system and the heat bath are illustrated in Fig. 1 in the main text. In the latter approach the total system starts in a product state of system $S$ (e.g. spin), bath $B$, and weight $W$, $\rho^{SBW} = \rho^S \otimes \tau^B \otimes \omega^W$, which together undergo *average energy conserving unitary evolution* with $V$:

$$\rho^{SBW} \longmapsto \sigma^{SBW} = V\, \rho^{SBW} V^\dagger. \tag{SI.13}$$

The assumption is that the total Hamiltonian is the sum of local terms, $H^{SBW} = H^S + H^B + H^W$. The average energy conservation constraint then reads $\mathrm{Tr}[\,(\sigma^{SBW} - \rho^{SBW})\, H^{SBW}] = 0$ and the average work extracted to the work storage system, $\langle W\rangle$, is identified with

$$\mathrm{Tr}[\,(\sigma^{SBW} - \rho^{SBW})\, H^W\,] = \langle W\rangle. \tag{SI.14}$$

Both the implicit and the explicit treatment of work are equivalent in the sense that the results obtained in one language can be translated in the other and vice versa. In particular, the implicit description used in this text[2] has an equivalent explicit formulation[11].

In the next section we will discuss single-shot extractable work in a projection process. One possibility to define work in this context is to chose the explicit work storage system as a 'work qubit' with a specific energy gap which has to be in a pure energy eigenstate before and after the protocol[12]. This way it is guaranteed that full knowledge about its state is present at all times and the work is stored in an ordered form. In this scenario the allowed unitary operations $V$ on the whole system $SBW$ have to conserve the energy exactly, not only on average, which amounts to $[V, H^{SBW}] = 0$.

## C Single-shot analysis

Instead of performing a thermodynamic process on an ensemble of $N$ identical and independent copies one can consider a single run of the process. Two major recent frameworks[4,12] have been developed to describe the optimal work that can be drawn from a system in a single run. The proposal by Åberg[4], involves changes of the Hamiltonian and identifies work with the deterministic energy change of the system when undergoing a unitary process. The proposal by Horodecki-Oppenheim[12], is formulated in terms of *thermal operations*[15], where work is associated with raising a two-level system, called the 'work qubit', with energy gap $W$ deterministically from the ground to the excited state.

However, when attempting to apply these two frameworks to find the single-shot work for the energy projections $\rho \to \eta^H$ captured by Eq. (1) in the main text one encounters an obstacle: both frameworks only apply to processes between initial and final states that are classical, i.e. states that are diagonal in the energy basis. Åberg discusses coherences in a separate framework[16], which does however not cover single-shot work extraction and only focusses on average quantities, similar to those in other references[2,11]. Horodecki-Oppenheim suggest that quantum states with coherences with respect to the energy eigenbasis are first decohered before applying the single-shot protocol. As discussed, apart from decohering there are other thermodynamic projection processes that map the initial state with coherences, $\rho$, to the final state $\eta^H = \sum_k \Pi_k^H\, \rho\, \Pi_k^H$ without coherences, where $\Pi_k^H$ are the projectors on the energy eigenstates of the Hamiltonian, $H$. Eq. (1) shows that the average work extracted in an optimal thermodynamic projection process is strictly positive while the decoherence process has zero work. Therefore one may expect a positive optimal work for projections also in the single-shot setting, with decohering a suboptimal choice, see Fig. SI.2.

Since our focus here is the $N \to \infty$ limit we will not aim to construct the single-shot case. Instead, to establish a notion of consistency between the average analysis and previous single-shot work results we consider the sequence $(\rho, H) \xrightarrow{a} (\rho_1, H) \xrightarrow{b} (\tau^H, H) \xrightarrow{c} (\eta^H, H)$ in which the Hamiltonian before and after each step are the same and $\rho_1$ is the rotated state defined above, Eq. (SI.7). Here $\tau^H = \frac{e^{-\beta H}}{Z}$ is the thermal state for Hamiltonian $H$ at inverse temperature $\beta = \frac{1}{k_B T}$ and $Z = \mathrm{Tr}[e^{-\beta H}]$ is the partition function. Step $a$ of this sequence rotates the initial non-diagonal state $\rho$ to the diagonal state $\rho_1$. As discussed, it cannot be treated with

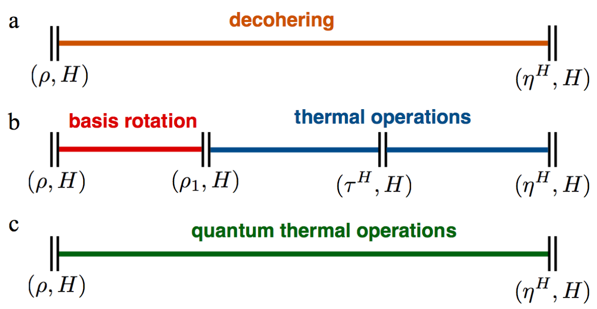


**Figure SI.2: Illustration of possible quantum thermodynamic processes that transform $(\rho, H)$ to $(\eta^H, H)$. a,** Decohering the state in the energy basis extracts no work. **b,** To perform a consistency check between the average and single-shot results it is possible to split the process into a basis rotation to $(\rho_1, H)$ with unknown single-shot work, but known average work, and two thermal operations that pass through the thermal state $(\tau^H, H)$ and are treatable in the single-shot framework[12]. **c,** General quantum thermodynamic processes could allow coherences and need not pass through intermediate fixed states.

the single-shot framework[4,12] but it is possible to associate an average extracted work with this unitary process, $\langle W^{(a)}\rangle = \mathrm{Tr}[(\rho - \rho_1)\, H]$. A single-shot analysis according to Horodecki-Oppenheim[12] can then be performed for the diagonal steps $b$ and $c$. This is possible because the steps go via the thermal state $\tau^H$. Step $b$ brings $\rho_1$ to $\tau^H$ and allows the extraction of the single-shot work[12]

$$W^{(b)} = k_B\, T\, \ln 2\, D^{\varepsilon}_{\min}(\rho_1 || \tau^H), \tag{SI.15}$$

where $D^{\varepsilon}_{\min}$ is the smooth min-relative entropy[17] and $\varepsilon \geq 0$ is the allowed failure probability of the process. Similarly, in Step $c$ the final state $\eta^H$ is formed from the thermal state by applying a protocol that costs work. This work is[12]

$$W^{(c)} = -k_B T\, \ln 2\, D^{\varepsilon}_{\max}(\eta^H || \tau^H), \tag{SI.16}$$

where $D^{\varepsilon}_{\max}$ is the smooth max-relative entropy[17]. In total, the single-shot work associated to Steps $b$ and $c$ of the process is $W^{(b)} + W^{(c)} = k_B T\, \ln 2\, (D^{\varepsilon}_{\min}(\rho_1||\tau^H) - D^{\varepsilon}_{\max}(\eta^H||\tau^H))$ with failure probability at most $2\varepsilon - \varepsilon^2 \approx 2\varepsilon$, when $\varepsilon$ is small.

To show consistency we now consider the average expected work extracted per copy if the single-shot protocol is carried out on $N \to \infty$ i.i.d. copies of the system. In such a calculation the work computed is an average value which is why $\langle W^{(a)}\rangle$, the average work contribution of the basis rotation in Step $a$, can be taken into account too. One obtains a total average work per copy of

$$\begin{aligned}
\langle W \rangle &= \langle W^{(a)}\rangle + k_B T\, \ln 2 \lim_{\varepsilon\to 0} \lim_{N\to\infty} \frac{1}{N} \left[ D^{\varepsilon}_{\min}\big(\rho_1^{\otimes N} || (\tau^H)^{\otimes N}\big) - D^{\varepsilon}_{\max}\big((\eta^H)^{\otimes N} || (\tau^H)^{\otimes N}\big) \right] \\
&= \langle W^{(a)}\rangle + k_B T\, \ln 2 \left[ D(\rho_1 || \tau^H) - D(\eta^H || \tau^H) \right] \\
&= \langle W^{(a)}\rangle + \mathrm{Tr}[\rho_1\, H] + \ln Z - k_B T\, S(\rho_1) - \mathrm{Tr}[\eta^H\, H] - \ln Z + k_B T\, S(\eta^H) \\
&= k_B T\, (S(\eta^H) - S(\rho)) \\
&\equiv \langle W_{\mathrm{opt}} \rangle,
\end{aligned} \tag{SI.17}$$

where we have used the quantum asymptotic equipartition theorem for relative entropies[18,19] in the second line. $D(\cdot||\cdot)$ is the standard quantum relative entropy defined by $D(\eta^H||\tau^H) = \mathrm{Tr}[\,\eta^H(\log \eta^H - \log \tau^H)\,]$ and likewise for $\rho_1$, where log is the logarithm to base 2. The quantities $D^{\varepsilon}_{\min}$ and $D^{\varepsilon}_{\max}$ as well as their regularized version, the standard quantum relative entropy $D$, can be seen as different measures characterizing the distance between two states. When applied here, they measure the 'distance' between the thermal state $\tau^H$ and another diagonal state in such a way that the operational meaning of this distance is given by the work one has to invest or is able to extract when transforming one into the other.

The derivation shows that in the asymptotic limit the optimal average work is recovered from the single-shot components. But it is important to realise that from Eq. (SI.17) one cannot conclude that the above single-shot process forming $\eta^H$ from $\rho_1$ is optimal. Going via the thermal state is just one option which is particularly convenient in this case as the processes of maximal work extraction and work of formation from the thermal state have been treated in the single-shot scenario[12]. It is an open question whether there are better single-shot protocols for general thermodynamic transformations, see Fig. SI.2b & SI.2c. The introduction of "catalysts" in single-shot thermodynamics[20] provides a promising avenue to establish bounds on the work that can be drawn from a state with coherences during a projection in the single-shot setting.

After making public our results on average work associated with removing coherences in thermodynamic projection processes very recently a paper appeared[21] that derives the work that can be extracted when removing

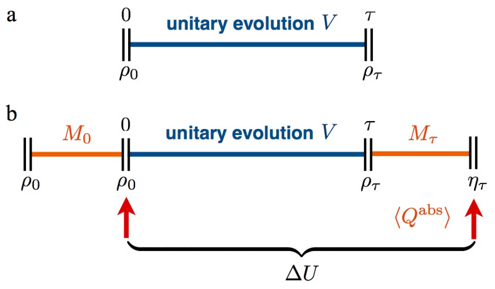


**Figure SI.3: Dynamical steps in a quantum fluctuation experiment. a,** The quantum Jarzynski relation is described as characterising the non-equilibrium work of processes that start in a thermal state $\rho_0$ and evolve unitarily ($V$), driven by a changing Hamiltonian, reaching the final state $\rho_\tau$ at time $\tau$. This unitary process has no heat contribution. **b,** Illustration of three steps that are assumed in the mathematical derivation of the quantum Jarzynski relation[6,22]: an initial energy measurement of $H^{(0)}$ indicated by $M_0$, the unitary evolution, and a final energy measurement of $H^{(\tau)}$ indicated by $M_\tau$. The ensemble state evolves here from $\rho_0$ to $\rho_\tau$ and then to $\eta_\tau$, the state $\rho_\tau$ with its coherences removed. The *observed* average energy difference $\Delta U = U(\eta_\tau) - U(\rho_0)$ encompasses both, the unitary process and the second projection process, and can in general contain a heat contribution $\langle Q^{\rm abs}\rangle$, in contrast to **a**.

coherences in a single-shot setting. In this paper the previously mentioned framework describing the catalytic role of coherence in thermodynamics by Åberg[16] is used together with insights from reference frames in quantum information theory. These results are in agreement with our findings and strengthen our conclusion that coherences are a fundamental feature distinguishing quantum from classical thermodynamics.

## D Quantum work fluctuation relation

A common route of deriving the quantum Jarzynski equation is as follows[5,6,22]. A quantum system is initialised in a thermal state $\rho_0 = \sum_n e^{-\beta(E_n^{(0)} - F_T^{(0)})}\, \Pi_n^{(0)}$ for a given Hamiltonian $H^{(0)} = \sum_n E_n^{(0)}\, \Pi_n^{(0)}$, with energy eigenvector projectors $\Pi_n^{(0)} = |e_n^{(0)}\rangle\langle e_n^{(0)}|$, at given inverse temperature $\beta = 1/(k_B T)$. Here $F_T^{(0)} = -k_B T \ln\left(\sum_n e^{-\beta E_n^{(0)}}\right)$ is the initial free energy associated with the initial Hamiltonian $H^{(0)}$. The aim is to calculate the average exponentiated work, $\langle e^{\beta W}\rangle$, that the quantum system will exchange when undergoing a unitary process $V$ that is generated by varying the Hamiltonian in time, i.e. $H^{(t)}$, from $H^{(0)}$ to a final $H^{(\tau)} = \sum_m E_m^{(\tau)}\, \Pi_m^{(\tau)}$. The final state after the unitary is the non-equilibrium state $\rho_\tau := V\, \rho_0\, V^\dagger$, see Fig. SI.3a.

To identify the work for an individual run of the experiment the energy of the system is measured at the beginning, by projecting into $\Pi_n^{(0)}$, and at the end, by projecting in the final energy basis $\Pi_m^{(\tau)} = |e_m^{(\tau)}\rangle\langle e_m^{(\tau)}|$. The (extracted) fluctuating work $W$ identified with each transition $|e_n^{(0)}\rangle \to |e_m^{(\tau)}\rangle$ is the (negative) observed *fluctuating energy difference* of the system

$$\Delta E = E_m^{(\tau)} - E_n^{(0)} = -W. \tag{SI.18}$$

The average exponentiated work then becomes

$$\langle e^{\beta W}\rangle = \sum_{m,n} e^{-\beta(E_m^{(\tau)} - E_n^{(0)})}\, p_{m,n}^{\tau}, \tag{SI.19}$$

where $p_{m,n}^{\tau}$ are the transition probabilities for energy jumps starting in $|e_n^{(0)}\rangle$ and ending in $|e_m^{(\tau)}\rangle$ at time $\tau$. These probabilities are given by

$$p_{m,n}^{\tau} = \mathrm{Tr}[\,\Pi_m^{(\tau)}\, V\, \Pi_n^{(0)}\, \rho_0\, \Pi_n^{(0)}\, V^\dagger\, \Pi_m^{(\tau)}\,] = e^{-\beta(E_n^{(0)} - F_T^{(0)})}\, \mathrm{Tr}[\,\Pi_m^{(\tau)}\, V\, \Pi_n^{(0)}\, V^\dagger\,], \tag{SI.20}$$

simplifying the exponentiated average work to

$$\langle e^{\beta W}\rangle = e^{\beta F_T^{(0)}} \sum_m e^{-\beta E_m^{(\tau)}} \sum_n \mathrm{Tr}[\,\Pi_m^{(\tau)}\, V\, \Pi_n^{(0)}\, V^\dagger\,]. \tag{SI.21}$$

The completeness of the projectors, $\sum_n \Pi_n^{(0)} = \mathbb{1}$, now finally results in the well-known quantum Jarzynski work relation

$$\langle e^{\beta W}\rangle = e^{\beta F_T^{(0)}} \sum_m e^{-\beta E_m^{(\tau)}} = e^{-\beta \Delta F}, \tag{SI.22}$$

where $\Delta F = F_T^{(\tau)} - F_T^{(0)}$ is the difference of the equilibrium free energies corresponding to the final and initial Hamiltonians, i.e. $F_T^{(\tau)} = -k_B T \ln\left(\sum_m e^{-\beta E_m^{(\tau)}}\right)$. Similarly, the average work extracted from the system is the average energy difference between $\rho_0$ and $\rho_\tau$

$$\begin{aligned} \langle W^{\text{unitary}} \rangle &= -\sum_{m,n} (E_m^{(\tau)} - E_n^{(0)})\, p_{m,n}^\tau = -\sum_m E_m^{(\tau)} p_m^\tau + \sum_n E_n^{(0)} p_n^0 && \text{(SI.23)} \\ &= -\text{Tr}[H^{(\tau)} \rho_\tau] + \text{Tr}[H_n^{(0)} \rho_0] = -U(\rho_\tau) + U(\rho_0) && \text{(SI.24)} \end{aligned}$$

where $p_m^\tau := \sum_n p_{m,n}^\tau = \text{Tr}[\rho_\tau \, \Pi_m^{(\tau)}]$ are the probabilities to find energies $E_m^{(\tau)}$ when a measurement of $H^{(\tau)}$ is performed on $\rho_\tau$. By construction, the initial probabilities to find energies $E_n^{(0)}$ are just the thermal probabilities, $p_n^0 := \sum_m p_{m,n}^\tau = \text{Tr}[e^{-\beta(E_n^{(0)} - F_T^{(0)})} \, \Pi_n^{(0)}] = e^{-\beta(E_n^{(0)} - F_T^{(0)})}$.

The derivations of the average work, $\langle W^{\text{unitary}} \rangle = U(\rho_0) - U(\rho_\tau)$, as well as the average exponentiated work, i.e. the quantum Jarzynski equality, $\langle e^{\beta W} \rangle = e^{-\beta \Delta F}$, are based on Eq. (SI.18) which was made assuming that the initial and final state of the process that is being characterised are $\rho_0$ and $\rho_\tau$. There is no question that the mathematical details of the derivations of the above relation are sound. Experimentally, there is however a need to acquire knowledge of the fluctuating energy to quantify the work and this requires the implementation of the second measurement, see Fig. SI.3b. Only after the measurement has been made can theoretical predictions be tested. The measurement is an unavoidable non-unitary component of the overall experimental process. Specifically, the ensemble state after the unitary, $\rho_\tau$, is further altered by the measurement to result in the final state $\eta_\tau$, i.e. it is the state $\rho_\tau$ with any coherences in the energy basis of $H^{(\tau)}$ removed.

While the experimentally observed average energy difference is not affected by the measurement step, i.e. $U(\eta_\tau) - U(\rho_0) = U(\rho_\tau) - U(\rho_0)$, the entropy difference does change, i.e. $S(\eta_\tau) - S(\rho_0) \neq S(\rho_\tau) - S(\rho_0) = 0$. This means that the system may absorb heat, $\langle Q^{\text{abs}} \rangle$, during the measurement step, indicated in Fig. SI.3b. Its actual value depends on *how* the measurement is conducted with the optimal heat positive, $\langle Q_{\text{opt}}^{\text{abs}} \rangle = k_B\, T(S(\eta_\tau) - S(\rho_\tau)) \geq 0$. Since $\Delta U = \langle Q^{\text{abs}} \rangle - \langle W \rangle$ (T1 in main text) this implies that in an experimental implementation of the Jarzynski relation the work done by the system on average can be more than previously thought, with the optimal value being $\langle W_{\text{opt}} \rangle = \langle W^{\text{unitary}} \rangle + k_B\, T\, (S(\eta_\tau) - S(\rho_\tau))$. In the special case that the average heat $\langle Q^{\text{abs}} \rangle$ is zero it is possible (although not necessary) that Eq. (SI.18), and thus the standard Jarzynski expression $\langle e^{\beta W} \rangle = e^{-\beta \Delta F}$, are correct. In particular this applies to classical measurements. We conclude that the suitability of identifying $W = -\Delta E$, and hence the validity of the quantum Jarzynski work relation depends on the details of the physical process that implements the measurement.

Quantum work fluctuation relations that have only one measurement[23,24], instead of the two discussed above, offer a feasible route of measuring work fluctuations experimentally. Instead of measuring separately the initial and final fluctuating energies, $E_n^{(0)}$ and $E_m^{(\tau)}$, to establish their joint probabilities, this method acquires *only* knowledge of the joint probabilities by measuring energy differences $\Delta E$ directly. But also here is one final measurement, in general on a non-diagonal state, needed.

## E Lower bound on entropy change

The entropy change during a projection with projectors $\{\Pi_k^{\mathcal{P}} = |\phi_k\rangle\langle\phi_k|\}_k$ can be lower bounded. In the following, $\|B\|_2 = \sqrt{\text{Tr}[B^\dagger B]}$ denotes the Hilbert-Schmidt norm of a linear operator $B$ acting on a $d$-dimensional Hilbert space describing the quantum system of interest. The lower bound reads[25]

$$\Delta S^{\mathcal{P}} := S(\eta^{\mathcal{P}}) - S(\rho) \geq \frac{1}{2} \left\| \rho - \frac{\mathbb{1}}{d} \right\|_2^2 \Delta A^{\mathcal{P}}. \qquad \text{(SI.25)}$$

Here, $S$ is the von Neumann entropy, $\rho$ the initial state and $\eta^{\mathcal{P}} = \sum_k \Pi_k^{\mathcal{P}} \, \rho \, \Pi_k^{\mathcal{P}}$ the final state after the projection process. Furthermore, $\Delta A^{\mathcal{P}}$ is the second smallest eigenvalue of the matrix $\mathbb{1} - M^T M$ where $M$ is the doubly stochastic matrix given by the entries $M_{kl} = |\langle \phi_k | \, l \, \rangle|^2$ and $\{| \, l \, \rangle\}_l$ is the eigenbasis of the initial state $\rho$.

Considering the two main terms on the right hand side of Eq. (SI.25) separately, $\|\rho - \mathbb{1}/d\|_2^2$ and $\Delta A^{\mathcal{P}}$, it becomes apparent that they characterise different properties of the initial state. The first term measures the distance of $\rho$ to the fully mixed state, $\mathbb{1}/d$, and quantifies the purity of $\rho$. It is maximal for all pure initial states and zero if and only if $\rho = \mathbb{1}/d$. In the special case of a spin-1/2 system it can be directly related to the length of the Bloch vector describing $\rho$ in the Bloch representation, a link that will be established below. The second term, $\Delta A^{\mathcal{P}}$, is related to the overlap of the eigenbasis of $\rho$, $\{| \, l \, \rangle\}_l$, and the projective basis, $\{|\phi_k\rangle\}_k$. It is zero if they are the same and maximal if they are mutually unbiased[26,27]. This can be seen as follows: if the two bases are the same, then the matrix $M$ is a permutation and consequently $M^T M$ is the identity. In this case, $\mathbb{1} - M^T M$ is the zero matrix and thus $\Delta A^{\mathcal{P}} = 0$. If $\{|\phi_k\rangle\}_k$ and $\{| \, l \, \rangle\}_l$ are mutually unbiased, i.e. if they fulfil $|\langle \phi_k | \, l \, \rangle|^2 = 1/d$ for all $k, l$, the matrix $M$ and thus also $M^T M$ is a rank-1 projector onto the space spanned by the

vector $(1,\dots,1)^T$. Hence, $\mathbb{1}-M^TM$ has eigenvalues $\{0,1,\dots,1\}$. One finds that the second largest eigenvalue is $\Delta A^{\mathcal{P}}=1$, which is also the maximal eigenvalue the matrix $\mathbb{1}-M^TM$ can have[28].

In the special case of the spin-1/2 system shown in Fig. SI.1a, the bound reads $\Delta S^H(\rho)\geq\frac{1}{4}\,|\vec{s}_\rho|^2\,\sin^2\theta$, where $\vec{s}_\rho$ is the Bloch vector of the initial state and $\theta$ is the angle between the eigenbasis of $\rho$, $\{|0\rangle,|1\rangle\}$, and the projective energy basis, $\{|e_0\rangle,|e_1\rangle\}$. Let $\rho=a\,|0\rangle\langle 0|+(1-a)\,|1\rangle\langle 1|$ be the initial state of the qubit. Furthermore, let $\eta^H=p\,|e_0\rangle\langle e_0|+(1-p)\,|e_1\rangle\langle e_1|$ be the final state after the energy projection, where $p=\mathrm{Tr}[\,|e_0\rangle\langle e_0|\,\rho]$ is the probability to obtain $|e_0\rangle$. As argued in Section SI A w.l.o.g. we can assume that $a\geq\frac{1}{2},p\geq\frac{1}{2}$. In the Bloch representation one can write $\rho=\frac{1}{2}(\mathbb{1}+\vec{s}\cdot\vec{\sigma})$ and $\eta^H=\frac{1}{2}(\mathbb{1}+\vec{t}\cdot\vec{\sigma})$. Here we used a different notation for the Bloch vectors of $\rho$, $\vec{s}:=\vec{s}_\rho$, and $\eta^H$, $\vec{t}:=\vec{s}_\eta$, for readability. The Pauli matrices are self-adjoint and fulfil $\mathrm{Tr}[\sigma_i\sigma_j]=2\delta_{ij}$. Hence we find

$$\left\|\rho-\frac{\mathbb{1}}{2}\right\|_2^2=\mathrm{Tr}\left[\left(\frac{1}{2}\vec{s}\cdot\vec{\sigma}\right)\left(\frac{1}{2}\vec{s}\cdot\vec{\sigma}\right)\right]=\sum_{i,j=1}^{3}\frac{1}{4}s_is_j\mathrm{Tr}[\sigma_i\sigma_j]=\frac{1}{2}|\vec{s}|^2, \tag{SI.26}$$

where $|\cdot|$ is the Euclidean metric in $\mathbb{R}^3$. This proves the form of the first factor in the bound. For the factor $\Delta A^H$ notice that by assumption $a\geq\frac{1}{2},p\geq\frac{1}{2}$ and thus we can write $|e_0\rangle\langle e_0|=\frac{1}{2}(\mathbb{1}+\frac{\vec{t}}{|\vec{t}|}\cdot\vec{\sigma})$ and $|0\rangle\langle 0|=\frac{1}{2}(\mathbb{1}+\frac{\vec{s}}{|\vec{s}|}\cdot\vec{\sigma})$. Therefore

$$|\langle e_0|0\rangle|^2=\mathrm{Tr}[|0\rangle\langle 0||e_0\rangle\langle e_0|]=\frac{1}{4}\mathrm{Tr}\left[\mathbb{1}+\frac{s_i}{|\vec{s}|}\sigma_i+\frac{t_i}{|\vec{t}|}\sigma_i+\frac{s_it_j}{|\vec{s}||\vec{t}|}\sigma_i\sigma_j\right]=\frac{1}{4}\left[2+0+0+2\frac{\vec{s}\cdot\vec{t}}{|\vec{s}||\vec{t}|}\right]=\frac{1}{2}[1+\cos\theta], \tag{SI.27}$$

where summations over double indeces are assumed and $\theta$ is the angle between the two Bloch vectors of $\rho$ and $\eta^H$, see Fig. SI.1a. By using orthonormality of both bases $\{|l\rangle\}_l$ and $\{|e_k\rangle\}_k$ it is shown that in the qubit case the matrices $M$ and $M^TM$ have the form

$$M=\begin{pmatrix}|\langle e_0|0\rangle|^2 & |\langle e_0|1\rangle|^2\\ |\langle e_1|0\rangle|^2 & |\langle e_1|1\rangle|^2\end{pmatrix}=\frac{1}{2}\begin{pmatrix}1+\cos\theta & 1-\cos\theta\\ 1-\cos\theta & 1+\cos\theta\end{pmatrix}\quad\text{and}\quad M^TM=\frac{1}{2}\begin{pmatrix}1+\cos^2\theta & 1-\cos^2\theta\\ 1-\cos^2\theta & 1+\cos^2\theta\end{pmatrix}. \tag{SI.28}$$

Computing the eigenvalues $\{\lambda_1,\lambda_2\}$ of $M^TM$ yields $\lambda_1=1$ and $\lambda_2=\cos^2\theta$. Therefore $\Delta A^H=1-\lambda_2=1-\cos^2\theta=\sin^2\theta$. In total, this concludes the proof of the bound for the special case of a spin-1/2 system when projected in the energy eigenbasis $\{\Pi_k^H\}_k=\{|e_0\rangle,|e_1\rangle\}$,

$$\Delta S^H(\rho)\geq\frac{1}{4}\,|\vec{s}_\rho|^2\,\sin^2\theta. \tag{SI.29}$$

To further illustrate the bound consider the special case when the initial state $\rho$ is pure and its eigenbasis mutually unbiased with respect to the energy eigenbasis, $\{|e_0\rangle,|e_1\rangle\}$. In this case the final state after the projection, $\eta^H$, is maximally mixed and we find

$$\Delta S^H(\rho)=S(\eta^H)-S(\rho)=\ln 2-0=\ln 2\approx 0.69\,. \tag{SI.30}$$

Here, the lower bound is equal to $\frac{1}{4}=0.25$ because $|\vec{s}_\rho|=1$ for a pure state $\rho$ and $\sin^2\theta=1$ for mutually unbiased bases. Thus in this example the bound is not particularly tight.

## F Access to correlated auxiliary systems

Similarly to erasure with a correlated memory[29] one can consider projections on a system $S$ that is correlated with an ancilla $A$ the experimenter has access to. Assuming a total Hamiltonian $H^{SA}=H^S\otimes\mathbb{1}^A+\mathbb{1}^S\otimes H^A$, we denote the global initial state by $\rho^{SA}$ and its marginals on $S$ and $A$ by $\rho^S=\mathrm{Tr}_A[\rho^{SA}]$ and $\rho^A=\mathrm{Tr}_S[\rho^{SA}]$, respectively.

**A note on notation.** For clarity we employ a slightly different notation here. The roles of initial state $\rho$ and final state $\eta$ are the same as in the main text and the previous sections of the Supplement. However, now the superscripts of the final state $\eta$ no longer denote the projection basis but the system for which $\eta$ describes the state. For instance, $\eta^S$ denotes the reduced state after the projection on system $S$ alone. The same holds for the superscript of the initial state, $\rho^{SA},\rho^S$ and $\rho^A$, and the Hamiltonians $H^{SA},H^S$ and $H^A$. Only the superscript $\mathcal{P}$ of the mutually orthogonal rank-1 projectors $\{\Pi_k^{\mathcal{P}}\}_k$ acting on system $S$ is kept to indicate which basis is being projected in.

For an initial global state $\rho^{SA}$ of system and ancilla a local projection map on $S$ results in a new global state

$$\eta^{SA} = \sum_k \left(\Pi_k^{\mathcal{P}} \otimes \mathbb{1}^A\right) \rho^{SA} \left(\Pi_k^{\mathcal{P}} \otimes \mathbb{1}^A\right). \tag{SI.31}$$

Due to the properties of the projectors the marginal state on $A$ is unchanged,

$$\eta^A = \mathrm{Tr}_S[\eta^{SA}] = \sum_k \mathrm{Tr}_S[(\Pi_k^{\mathcal{P}} \otimes \mathbb{1}^A)\, \rho^{SA}\, (\Pi_k^{\mathcal{P}} \otimes \mathbb{1}^A)] = \sum_k \mathrm{Tr}_S[\rho^{SA}\, (\Pi_k^{\mathcal{P}} \otimes \mathbb{1}^A)] = \mathrm{Tr}_S[\rho^{SA}] = \rho^A. \tag{SI.32}$$

The reduced state of the system becomes $\eta^S \equiv \mathrm{Tr}_A[\eta^{SA}] = \sum_k \Pi_k^{\mathcal{P}}\, \rho^S\, \Pi_k^{\mathcal{P}} = \sum_k p_k\, \Pi_k^{\mathcal{P}}$ where $p_k = \mathrm{Tr}[\rho^S \Pi_k^{\mathcal{P}}]$, and the conditional states on $A$ after the process are denoted $\eta_k^A = p_k^{-1}\, \mathrm{Tr}_S[\, (\Pi_k^{\mathcal{P}} \otimes \mathbb{1}^A)\, \rho^{SA}\, (\Pi_k^{\mathcal{P}} \otimes \mathbb{1}^A)\,]$ for all $k$. The global entropy change associated with the local projection is

$$\begin{aligned}\Delta\mathcal{S}^{\mathcal{P}} &= S(\eta^{SA}) - S(\rho^{SA}) = S(\{p_k\}) + \sum_k p_k S(\eta_k^A) - S(\rho^{SA}) = S(\eta^S) - S(\rho^S) + S(\rho^S) + \sum_k p_k S(\eta_k^A) - S(\rho^{SA}) \\ &= \Delta S^{\mathcal{P}} + \delta^{\mathcal{P}}(A:S).\end{aligned} \tag{SI.33}$$

In the second equality it was used that $\eta^{SA}$ is a classical-quantum-state and $S(\{p_k\}) = -\sum_k p_k \ln p_k$ stands for the classical Shannon entropy[30] which is equal to the von Neumann entropy of $\eta^S$ because the final state on $S$ is a classical mixture of states from the projective basis. Here we defined a measure of correlations between the ancilla and the system, $\delta^{\mathcal{P}}(A:S) = S(\rho^S) - S(\rho^{SA}) + \sum_k p_k S(\eta_k^A)$, related to the quantum discord. It depends on the projectors $\{\Pi_k^{\mathcal{P}}\}_k$ and is always positive[31,32]. Thus the entropy change of $SA$ can be bigger than the local entropy change, $\Delta S^{\mathcal{P}} = S(\eta^S) - S(\rho^S)$, on the system alone.

As is shown in the main text, Eq. (2), the optimal extractable work in a thermodynamic projection process on system $S$ alone is $\langle W_{\mathrm{opt}}\rangle = k_B\, T\, \Delta S^{\mathcal{P}} - \Delta U^{\mathcal{P}}$, where $\Delta S^{\mathcal{P}}$ is the entropy change of the system and $\Delta U^{\mathcal{P}}$ its change in internal energy. This result stays intact when generalizing to projections in the presence of ancillary systems if one takes the total changes of these quantities on $SA$ instead of the change on $S$ only. In the global process the total internal energy change is equal to the energy change of the system only as the local state of the ancilla is unchanged and the total Hamiltonian is the sum of local Hamiltonians. Thus using side information the overall optimal extractable work amounts to

$$\begin{aligned}\langle \mathcal{W}_{\mathrm{opt}}\rangle &= k_B T\, \Delta\mathcal{S}^{\mathcal{P}} - \Delta U^{\mathcal{P}} = k_B T\, \Delta S^{\mathcal{P}} + k_B T\, \delta^{\mathcal{P}}(A:S) - \Delta U^{\mathcal{P}} \\ &= \langle W_{\mathrm{opt}}\rangle + k_B T\, \delta^{\mathcal{P}}(A:S),\end{aligned} \tag{SI.34}$$

where $\langle W_{\mathrm{opt}}\rangle$ is the work of an optimal thermodynamic projection process without access to correlated systems, Eq. (2) in the main text.

Discord was first discussed in a thermodynamic context by Zurek[33], where he related it to the advantage a quantum Maxwell demon could have over a classical one. In general the quantum discord, $\delta(A:S)$, is defined as the minimum of $\delta^{\mathcal{P}}(A:S)$ over all sets of projectors $\{\Pi_k^{\mathcal{P}}\}_k$ whereas in our case this set is fixed (see e.g. Modi *et al.*[34] for a review). Therefore it is found that even for states with no quantum discord, usually referred to as classically correlated states, a difference in work associated with thermodynamic projection processes can be observed. This contrasts with the erasure process[29] where an advantage could only be gained for highly entangled states.

One may ask what global states on $SA$ maximize $\langle \mathcal{W}_{\mathrm{opt}}\rangle$ for a given state $\rho^S$ on $S$. Expectedly, it can be shown that purifications of $\rho^S$ yield the best improvement in terms of extracted work. Given $\rho^S = \sum_l a_l |l\rangle\langle l|$ any purification is, up to isometries on the purifying system[1], equivalent to $|\Psi\rangle = \sum_l \sqrt{a_l}|l\rangle^S|l\rangle^A$ for some orthonormal basis $\{|l\rangle^A\}_l$ of $A$. For such a state the conditional states on $A$ after the projection, $\rho_k^A$, are pure for all $k$ which implies that they have zero entropy. This implies

$$\delta^{\mathcal{P}}(A:S) = S(\rho^S) - S(|\Psi\rangle\langle\Psi|) + \sum_k p_k S(\eta_k^A) = S(\rho^S) - 0 + \sum_k p_k \cdot 0 = S(\rho^S). \tag{SI.35}$$

The optimal total extracted work from a purified state on $SA$ in a thermodynamic projection process is therefore $\langle \mathcal{W}_{\mathrm{opt}}\rangle = k_B T\, S(\eta^S) - \Delta U^{\mathcal{P}}$ which can be shown to be the maximum for fixed $\rho^S$ and projectors $\{\Pi_k^{\mathcal{P}}\}_k$. One way to see this is the Supplementary Lemma[35]:

**Supplementary Lemma.** *Let $\rho^{SA}$ be an arbitrary state on a bipartite system $SA$, and let $\{\Pi_k^{\mathcal{P}}\}_k$ be a complete set of rank-1 orthogonal projectors on $S$. Consider the global state after the projection, $\eta^{SA} = \sum_k (\Pi_k^{\mathcal{P}} \otimes \mathbb{1}^A)\, \rho^{SA}\, (\Pi_k^{\mathcal{P}} \otimes \mathbb{1}^A) = \sum_k p_k\, \Pi_k^{\mathcal{P}} \otimes \eta_k^A$, where $p_k = \mathrm{Tr}[(\Pi_k^{\mathcal{P}} \otimes \mathbb{1}^A)\, \rho^{SA}]$ are the probabilities to measure $k$ on $S$ and $\eta_k^A = p_k^{-1}\, \mathrm{Tr}_S[(\Pi_k^{\mathcal{P}} \otimes \mathbb{1}^A)\, \rho^{SA}\, (\Pi_k^{\mathcal{P}} \otimes \mathbb{1}^A)]$ are the conditional states on $A$. Then*

$$S(\rho^{SA}) \geq \sum_k p_k S(\eta_k^A). \tag{SI.36}$$

*Proof.* We model the process on $S$ as an isometry $\Phi^{S\to S\tilde{S}} = \sum_k |\psi_k\rangle^{\tilde{S}} \otimes \Pi_k^S$, where $\tilde{S}$ is a copy of $S$ and $\{|\psi_k\rangle^{\tilde{S}}\}_k$ is an orthonormal basis of $\tilde{S}$. The state after applying the isometry is denoted $\eta^{S\tilde{S}A} = \Phi\rho^{SA}\Phi^\dagger$ and we note that $\mathrm{Tr}_{\tilde{S}}[\eta^{S\tilde{S}A}] = \eta^{SA}$. Furthermore, isometries do not change (von Neumann) entropy and thus, $S(\eta^{S\tilde{S}A}) = S(\rho^{SA})$. In addition, by construction of $\tilde{S}$ the marginals of the final state on $S$ and $\tilde{S}$ have the same entropy: $S(\eta^{\tilde{S}}) = S(\eta^S)$. Since $S(\eta^{S\tilde{S}A}) \geq |S(\eta^{SA}) - S(\eta^{\tilde{S}})| \geq S(\eta^{SA}) - S(\eta^{\tilde{S}})$ (see e.g. Nielsen & Chuang[1]). Thus

$$S(\rho^{SA}) = S(\eta^{S\tilde{S}A}) \geq S(\eta^{SA}) - S(\eta^{\tilde{S}}) = S(\eta^{SA}) - S(\eta^S) = \sum_k p_k S(\eta_k^A), \tag{SI.37}$$

where in the last equality we made use of the fact that $\eta^{SA}$ is a classical-quantum state. □

Going back to Eq. (SI.33) and applying the the Supplementary Lemma we see that in general $\langle \mathcal{W}_{\text{opt}} \rangle = k_B T\, \Delta\mathcal{S}^{\mathcal{P}} - \Delta U^{\mathcal{P}} = k_B T\, \left[ S(\eta^S) + \sum_k p_k S(\eta_k^A) - S(\rho^{SA}) \right] - \Delta U^{\mathcal{P}} \leq k_B T\, S(\eta^S) - \Delta U^{\mathcal{P}}$, which proves that purifications on $SA$ yield the maximally possible extracted work.

## References


[1] Nielsen, M.A. & Chuang, I.L. *Quantum Computation and Quantum Information* Cambridge University Press, Cambridge UK (2000).

[2] Anders, J. & Giovanetti, V. Thermodynamics of discrete quantum processes. *New J. Phys.* **15,** 033022 (2013).

[3] Gemmer, J., Michel, M. & Mahler, G. *Quantum Thermodynamics* Springer (2009).

[4] Åberg, J. Truly work-like work extraction via a single-shot analysis. *Nat. Commun.* **4,** 1925 (2013)

[5] Morikuni, Y. & Tasaki, H. Quantum Jarzynski-Sagawa-Ueda Relations. *J. Stat. Phys.* **143,** 1 (2011).

[6] Talkner, P., Lutz, E. & P. Hänggi. Fluctuation theorems: Work is not an observable. *Phys. Rev. E* **75,** 050102 (R) (2007).

[7] Batalhao, T.B. *et al.* Experimental Reconstruction of Work Distribution and Study of Fluctuation Relations in a Closed Quantum System. *Phys. Rev. Lett.* **113,** 140601 (2014).

[8] Esposito, M., Lindenberg, K. & Van den Broeck, Ch. Entropy production as correlation between system and reservoir. *New J. Phys.* **12,** 013013 (2010).

[9] Seifert, U. Stochastic thermodynamics, fluctuation theorems and molecular machines. *Rep. Prog. Phys.* **75,** 126001 (2012).

[10] Saira, O-P., *et al.* Test of the Jarzynski and Crooks Fluctuation Relations in an Electronic System. *Phys. Rev. Lett.* **109**,180601 (2012).

[11] Skrzypczyk, P., Short, A.J. & Popescu, S. Work extraction and thermodynamics for individual quantum systems. *Nat. Commun.* **5,** 4185 (2014)

[12] Horodecki, M. & Oppenheim, J. Fundamental limitations for quantum and nanoscale thermodynamics. *Nat. Commun.* **4,** 2059 (2013)

[13] Pusz, W. & Woronowicz, S.L. Passive states and KMS states for general quantum systems. *Comm. Math. Phys.* **58,** 273 (1978).

[14] Lenard, A. Thermodynamical proof of the Gibbs formula for elementary quantum systems. *J. Stat. Phys* **19,** 575 (1978).

[15] Brandão, F.G.S.L., Horodecki, M., Oppenheim, J., Renes, J.M. & Spekkens, R.W. The Resource Theory of Quantum States Out of Thermal Equilibrium. *Phys. Rev. Lett.* **111,** 250404 (2013)

[16] Åberg, J. Catalytic Coherence. *Phys. Rev. Lett.* **113,** 150402 (2014)

[17] Datta, N. Min- and max-relative enropites and a new entanglement monotone. *IEEE Trans. Inf. Theory* **55,** 2816-2826 (2009)

[18] Tomamichel, M. *A framework for non-asymptotic quantum information theory.* PhD Thesis, ETH Zurich (2012)

[19] Dupuis, F., Kraemer, L., Faist, P., Renes, J.M. & Renner, R. Generalized Entropies. XVIIth International Congress on Mathematical Physics, 134-153 (2013).

[20] Brandão, F.G.S.L., Horodecki, M., Ng, N.H.Y., Oppenheim, J. & Wehner, S. The second laws of quantum thermodynamics. *PNAS* **112**, 3275 (2015).

[21] Korzekwa, K., Lostaglio, M., Oppenheim, J. & Jennings, D. The extraction of work from quantum coherence. Preprint at arXiv:1506.07875 (2015).

[22] Mukamel, S. Quantum extension of the Jarzynski relation: Analogy with stochastic dephasing. *Phys. Rev. Lett.* **90,** 170604 (2003).

[23] Mazzola, L., De Chiara, G. & Paternostro, M. Measuring the characteristic function of the work distribution. *Phys. Rev. Lett.* **110** 230602 (2013).

[24] Roncaglia, A., Cerisola, F. & Paz, J.P. Work measurement as a Generalized Quantum measurement. *Phys. Rev. Lett* **113** 250601(2014).

[25] Streater, R.F. Convergence of the Quantum Boltzmann Map. *Commun. Math. Phys.* **98,** 177 (1985).

[26] Appleby, D.M. SIC-POVMs and the extended Clifford group. *J. Math. Phys* **46,** 052107 (2005).

[27] Durt, T., Englert, B.-G., Bengtsson, I. & Zyczkowski, K. On mutually unbiased bases. *Int. J. Quantum Inform.* **08,** 535 (2010).

[28] Ando, T. Majorization, doubly stochastic matrices, and comparison of eigenvalues. *Linear Algebra Appl.* **118,** 163-248 (1989).

[29] del Rio, L., Åberg, J., Renner, R., Dahlsten, O.C.O. & Vedral, V. The thermodynamic meaning of negative entropy. *Nature* **474,** 61 (2011).

[30] Shannon, C.E. A Mathematical Theory of Communication. *Bell Syst. Tech. J.* **27,** (3) 379-423 (1948)

[31] Ollivier, H. & Zurek, W.H. Quantum discord: a measure of the quantumness of correlations. *Phys. Rev. Lett.* **88,** 017901 (2000).

[32] Henderson, L. & Vedral, V. Classical, quantum and total correlations. *J. Phys. A: Math. Gen.* **34,** 6899 (2001).

[33] Zurek, W.H. Quantum discord and Maxwell's demons. *Phys. Rev. A* **67,** 012320 (2003).

[34] Modi, K., Brodutch, A., Cable, H., Paterek, T. & Vedral, V. The classical-quantum boundary for correlations: Discord and related measures. *Rev. Mod. Phys.* **84,** 1655 (2012).

[35] Renner, R. Private Communication (2014).
